# Developing a spatio-temporal model for banana bunchy top disease: leveraging remote sensing and survey data

**DOI:** 10.3389/fpls.2025.1521620

**Published:** 2025-06-09

**Authors:** Renata Retkute, Christopher A. Gilligan

**Affiliations:** Epidemiology and Modelling Group, Department of Plant Sciences, University of Cambridge, Cambridge, United Kingdom

**Keywords:** epidemiological modeling, banana bunchy top virus, remote sensing, crop management, parameter estimation

## Abstract

Epidemics of Banana Bunchy Top Disease (BBTD) in sub-Saharan Africa are threatening global food security and endangering the livelihoods of smallholder farmers. This study introduces methods for developing data-based models to derive banana production maps and process-based models to assess the potential spread of BBTV at a landscape scale. We introduce two novel aspects: a methodology for deriving probabilistic banana production maps based on high-resolution remote sensing products and parameterization of the epidemiological model for BBTD from limited survey data. We generated a countrywide banana production map for Tanzania and a state-wide map for Ogun State in Nigeria. We used the banana map together with published data from BBTD surveys to parameterize a model for BBTD spread in Tanzania. Our results emphasize the importance of surveys, as having data on the presence and absence of Banana Bunchy Top Virus (BBTV) at different stages of epidemics is crucial not only for effective control of the disease but also for prediction, including making reasonable model assumptions, model parameterization, and model validation that underpin predictions.

## 1 Introduction

Bananas and plantains rank among the world’s top 10 food crops (Kumar et al., 2015). In East Africa, bananas serve as a staple food for over seven million people and are a primary source of income for millions of smallholder farmers (Gold et al., 2002). Over the past few decades, several significant banana diseases have emerged and spread extensively across different regions, posing a risk to food security and jeopardizing the livelihoods of smallholder farmers (Tatineni and Hein, 2023).

Banana bunchy top disease, caused by the banana bunchy top virus (BBTV: genus *Babuvirus*, family *Nanoviridae*), is the most destructive viral disease affecting bananas globally, posing a significant threat to smallholder banana cultivation. The virus is primarily transmitted by the aphid vector *Pentalonia nigronervosa* and further disseminated using infected propagation materials (Magee, 1927, 1940). Banana Bunchy Top Virus infection can result in yield losses of up to 100% (Okonya et al., 2019).

Over the past decade, BBTV has spread across sub-Saharan Africa, with confirmed cases in the Democratic Republic of Congo, Angola, Cameroon, Gabon, Malawi (Kumar et al., 2011), Nigeria (Adegbola et al., 2013), Benin (Lokossou et al., 2012), Togo (Kolombia et al., 2021), Uganda (Ocimati et al., 2021, 2024), and Tanzania (Shimwela et al., 2022). Recent findings confirm that BBTV has been established in Tanzania, with BBTV being found in 10 regions (Mahuku and Kumar, 2023). Urgent interventions are needed to halt the spread of the virus throughout the country.

Mathematical models of epidemics offer valuable insights into the mechanisms driving disease spread and enable comparison of different management strategies for viral spread. There are only a few large-scale spatially explicit models of plant disease spread, including sudden oak death (Cunniffe et al., 2016), *Xylella fastidiosa* in olive trees (White et al., 2017), citrus Huanglongbing disease (Mastin et al., 2020; Nguyen et al., 2023), cassava brown streak disease (Godding et al., 2023), and brown rot in peach (Radici et al., 2024). One important limiting factor is the absence of host maps upon which to model pathogen spread in the target countries.

We introduced a novel approach for generating banana cultivation maps using high-resolution remote sensing data. Our method involves identifying a sample of locations with banana cultivation through photo interpretation of high-resolution satellite imagery. We then created binary maps indicating the presence or absence of banana by utilizing high-resolution data on vegetation indices, canopy height, and built-up areas. Remote sensing and production data at the regional level were used to construct a banana production map of Tanzania. Recently, high-resolution RGB and multispectral aerial imagery from an unmanned aerial vehicle (UAV) have been deployed to identify bananas in smallholder farming systems in Ogun State, Nigeria (Alabi et al., 2022). We tested our proposed method against predictions based on UAV imagery. Finally, we used the host map for Tanzania together with limited published survey data from 2020 and 2023 to parameterize a model for the spread and transmission of BBTV at country-wide scales.

## 2 Material and methods

### 2.1 Constructing banana production map

We developed a novel method to derive banana production maps. The high-resolution remote sensing products used to generate host maps for Tanzania are summarized in **Table 1**. Tanzania is divided into 31 regions (administrative level 1) and 184 districts (administrative level 2). The Global Canopy Height Map dataset provides data worldwide for the period 2009–2020 (Tolan et al., 2024). We used information on canopy height to discriminate between objects such as trees, banana plants, shrubs, and crops. Sentinel-2 data were preprocessed using the Google Earth Engine platform (Gorelick et al., 2017). Specifically, we filtered cloudy pixels with a cloud percentage value larger than 5% and masked poor-quality surface reflectance values using the cloud mask (QA60) band. We extracted image collections dated from 1 August 2023 to 1 August 2024. For each available date, we calculated the Normalized Differential Vegetation Index (NDVI) as follows:

**Table 1:** Datasets used to derive banana production map.

Data type	Source (reference)	Format (resolution)	Size
Vegetation	Copernicus Harmonized Sentinel-2 MultiSpectral Instrument, Level-2A (Richter et al., 2012)	Raster (10 m resolution)	175.85 GB
Canopy height	The Meta Global Canopy Height Map (Tolan et al., 2024)	Raster (1 m resolution)	95.82 GB
Built-up areas	Google Open Buildings v3 (Sirko et al., 2021)	Polygons	6.2 GB
Protected areas	The World Conservation Monitoring Center’s (WCMC) Africa protected areas database (UNEP-WCMC, 2019)	Polygons	152 KB
Permanent water bodies	National Geospatial-Intelligence Agency Tanzania DCW Water Bodies (Hijmans, 2019)	Polygons	789 KB
Area planted to bananas	The Tanzania National Sample Census of Agriculture 2019–2020 (National Sample Census of Agriculture, 2019/2020)	Regional scale	2 KB


(1) NDVI=(NIR−Red)/(NIR+Red).

Here, *Red* and *NIR* are the spectral reflectance measurements acquired in the red (visible) and near-infrared regions, respectively. The NDVI varied between −1.0 and +1.0. We aggregated all the processed images into a median composite.

We derived a banana production map at a resolution of 1 km × 1 km. The map construction workflow involves the following steps:

1. We manually labeled 100 locations with banana production using photo-interpretation of high-resolution satellite imagery from Google Earth Pro (Wuthrich, 2006).
2. We extracted canopy height values and annual mean NDVI values with 5 m radius around the location. This provided a distribution of canopy heights (*F_CH_*) and NDVI values (*F_NDVI_*).
3. For each 1 km × 1 km grid cell, we created a 1 m × 1 m pixel subgrid.
4. We masked the protected areas and permanent water bodies for each 1 m × 1 m pixel.
5. We masked a 1 m × 1 m pixel if it intersected a building polygon.
6. For each unmasked 1 m × 1 m pixel, we extracted the values of the canopy height and annual mean NDVI.
7. We equated the lower and upper bounds with upper and lower interquartile values of canopy height and NDVI and used these to create a binary map for banana presence/absence at 1 m resolution.
8. We counted the number of 1 m × 1 m grid cells with bananas and divided them by the total number of grid cells to obtain the fraction of each 1 km × 1 km grid cell occupied by bananas.
9. We calibrated the map at the regional level using data on the total area planted with bananas (as monocultures plus mixed cropping) from the Tanzania National Sample Census of Agriculture 2019–2020 (National Sample Census of Agriculture, 2019/2020). We renormalized the fraction of 1 km × 1 km grid cells planted with bananas so that total area in the region was equal to the total area from the National Census as follows:
(2) $$f_{i}^{adj}=\frac{H_{k}}{\sum_{j=1}^{n_{k}}f_{j}^{calc}}f_{i}^{calc},$$
  where $f_{i}^{calc}$ is the calculated fraction, $H_{k}$ of total banana production in a region k , and $f_{i}^{adj}$ is the adjusted (normalized) fraction.
10. Steps 7–9 were repeated a hundred times to obtain a probabilistic map of banana production.

We also generated a banana production map for Ogun State in Nigeria. The study area referenced by Alabi et al. (2022) spans approximately 325 km^2^. For this, we used the same data sources for vegetation, canopy height, and built-up areas as outlined in **Table 1** but focused on the region encompassing Ogun State, with longitudes between 2.69 and 3.018 and latitudes between 6.56 and 6.97. Unlike the approach used to create a country-level banana production map for Tanzania, we applied a mask based on high-resolution oil palm production maps developed for West Africa (Descals et al., 2021).

### 2.2 Model formulation

The model uses a spatially explicit susceptible-infection framework that continuously monitors the infection status of each grid cell. The model allows for varying banana densities and pathogen entry routes while also considering localized increases in pathogen density at specific sites and virus spread between grid cells.

Each 1 km × 1 km grid cell *i* is described by banana density *h_i_*. The grid cells were divided into two sets: infected (*I*) and susceptible (*S*). A grid cell is susceptible if all banana plants in it are healthy. A susceptible grid cell becomes infected when the first banana plant in the grid cell becomes infected. Exposure can occur via either primary transmission (from the outside) or secondary transmission (between the grid cells).

Following the first infection, the pathogen spread within the grid cell is driven by a local, deterministic spread. To model the local infection within a grid cell, we employed a logistic equation to represent the progression of the fraction of infected hosts over time. Specifically, for an infected grid cell *i*, first infected at time *t_i_*, the density of infected hosts at any later time is given by:


(3) $$\rho_{i}\left(t\right)=\frac{1}{1+\left(\frac{1}{p_{0}}-1\right)e^{-r\left(t-t_{i}\right)}},$$

where *p*_0_ is prevalence at the first infection, and *r* is the logistic rate.

The BBTV exposure rate at which susceptible grid cell *i* becomes infected is:


(4) $$\lambda_{i}\left(t\right)=h_{i}\left(\epsilon +\beta \sum_{j\in I_{t},j\neq i}h_{j}\rho_{j}\left(t\right)K\left(\alpha ,d_{i,j}\right)\right),$$

where *ϵ* is the primary transmission rate, *β* is the secondary transmission rate; *I_t_*, is an index set of infectious grid cells at time *t*; *d_i,j_* is the Euclidean distance between the centers of grid cells *i* and *j*; and *K*(*d_i,j_*) is a dispersal kernel. We define the dispersal kernel as:


(5) $$K\left(\alpha ,d\right)=\frac{1}{2\pi \alpha^{2}}\text{exp }(-d/\alpha ).$$

Here *α* represents the dispersal scale. We simulated the process of infection according to the Gillespie algorithm (Gillespie, 1976, 1977), which accounts for time-inhomogeneous rates.

### 2.3 Parameter estimation

The parameter estimation was performed in two steps. First, we parameterized the logistic equation, which represents the progression of the fraction of infected hosts over time within a grid. For this task, we used the data published by Omondi et al. (2020). The data were obtained from an experiment on natural infection by BBTV, where monthly surveys were conducted by trained personnel. The data were derived from monoculture systems, which represent the upper limit of BBTD dynamics within a field. The logistic equation was fitted using maximum likelihood.

We used publicly available data for two time points to parameterize BBTV spread between grid cells. The first case of BBTV was reported in the Kigoma Region of Tanzania in December 2020 (Shimwela et al., 2022). Banana plants with typical BBTV symptoms (severe stunting, leaves with shortened petioles, chlorotic streaks, and yellow leaf margins) have been found in several banana fields in Muhinda and Mwayaya villages (Shimwela et al., 2022). Between May and July 2023, surveys were conducted across 85 districts in 15 regions (Mahuku and Kumar, 2023). Evidence of BBTV infection was observed in 22 districts. The introduction of BBTV into eastern Tanzania was attributed to the planting material purchased from a nursery, where the survey team detected BBTV symptomatic suckers (Mahuku and Kumar, 2023).

We developed a novel parameter estimation method by introducing a score metric that evaluates the similarity between the simulated outbreaks and survey data at the district level. This was dictated by the granularity of the survey data used for parameterization. To estimate the parameters for BBTV spread between grid cells, we used an Approximate Bayesian Computation (ABC) rejection technique (Minter and Retkute, 2019). The algorithm accepts the proposed parameter values based on the closeness of the simulated data to the observed data. Non-informative uniform priors are used for the parameters, *ϵ*, *β*, and *α*. For each sampled parameter set, we ran 100 individual simulations and calculated the fraction of simulations that resulted in grid cell *i* being infected, *f_i_*. We devised the following score metric to assess the similarity between observed and simulated outbreaks:


(6) $$M_{t}=\frac{1}{N_{D}}(\begin{array}{c} \sum_{k\in D_{P}\left(t\right)}\frac{1}{n_{k}}\left(\sum_{i=1}^{n_{k}}f_{i}\right)+\sum_{k\in D_{A}\left(t\right)}\frac{1}{n_{k}}\left(\sum_{i=1}^{n_{k}}(1-f_{i})\right)+\sum_{k\in D_{NS}\left(t\right)}\frac{1}{n_{k}}\left(\sum_{i=1}^{n_{k}}(1-f_{i})\right) \end{array}),$$

here *D_P_(t)* is a set of districts where BBTV was found at time *t*, *D_A_(t)* is a set of districts in which BBTV was not found at time *t*, and *D_NS_(t)* is a set of districts where no surveillance was conducted, *n_k_* is the number of grid cells in a district *k*, and *N_D_* is the number of districts. The metric score increased as many grid cells and many simulations produced a status corresponding to the survey results (i.e. presence or absence of BBTV in a district). For the districts where no surveillance was conducted, we gave preference for an absence of BBTV. A score of *M* = 1 corresponded with a parameter set that produced simulations which exactly reproduced survey results, i.e. *f_i_* = 1 for all grid cells in districts where BBTV was present, and *f_i_* = 0 for grid cells in other districts.The aim of the traditional ABC rejection algorithm is to minimise the distance between summary statistics of simulated and observed data (Minter and Retkute, 2019). Here we introduced a novel adaptation of the ABC rejection algorithm, where the aim is to maximise the score given by Equation 6.

## 3 Results

### 3.1 Banana production map

#### 3.1.1 Tanzania

**Figure 1:**
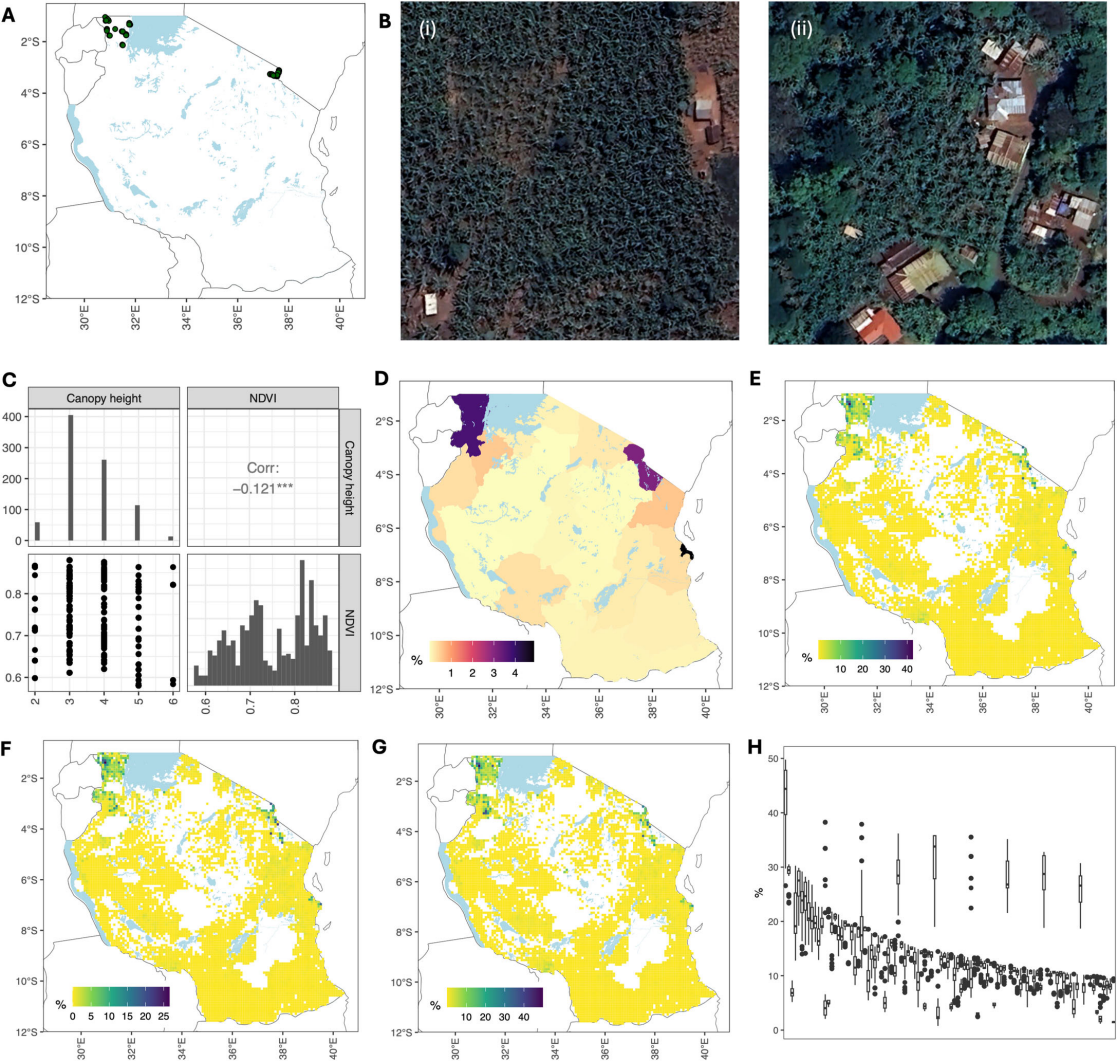
Constructing banana production map for Tanzania. **(A)** Locations used to obtain typical values of banana canopy height and NDVI (50 locations in Kagera and 50 locations in Kilimanjaro). **(B)** Two examples of locations: (i) Kagera region and (ii) Kilimanjaro region. **(C)** Distribution of canopy height (m) and NDVI values obtained from sampled locations in **(A)**. **(D)** Percentage of regional area planted with bananas (in monoculture and mixed cropping) from the Tanzania National Sample Census of Agriculture 2019–2020 (National Sample Census of Agriculture, 2019/2020). **(E)** Mean percentage of grid cells planted with bananas. **(F)** Minimum percentage of grid cells planted with bananas. **(G)** Maximum percentage of grid cells planted with bananas. **(H)** Boxplot of the percentage planted with bananas for 100 grid cells with the highest banana production. Grid cells were ranked according to the median values. Satellite images in **(B)** were obtained from the Google Earth Engine (Imagery @2024 Airbus, CNES/Airbus, Landsat/Copernicus, Maxar Technology).

After running a pilot study, we chose two regions to obtain typical values of banana canopy height and NDVI, Kagera and Kilimanjaro. According to the Tanzania National Sample Census of Agriculture 2019–2020, the Kagera region had the largest planted area with banana (1,371.8 km^2^), followed by Kilimanjaro (407.4 km^2^) (National Sample Census of Agriculture, 2019/2020). These two regions account for 56% of the bananas planted in mainland Tanzania. We identified 50 locations in each of the two regions using photointerpretation of high-resolution satellite imagery from Google Earth Pro (Wuthrich, 2006). These locations are shown in **Figure 1A**. Examples of high-quality images with well-defined banana canopies are shown in **Figure 1B**. The example from the Kagera region shows an area planted exclusively with banana (**Figure 1Bi**), whereas the example from the Kilimanjaro region shows bananas growing in a smallholder plot together with several different types of trees (**Figure 1Bii**). Histograms and bivariate distributions corresponding to canopy height and annual mean NDVI (Equation 1) within a radius of 5 m from the chosen locations are shown in **Figure 1C**. The range for canopy height was between 2 m and 6 m, and the annual median NDVI was in the range of ∈ [0.55,0.88]. The values for canopy height agreed with those of other studies (zum Felde et al., 2016).

The algorithm performed well in regions with a high banana presence, such as Kagera, Kilimanjaro, Dar es Salaam, Geita, and Mwanza (**Supplementary Figure A1**). However, it overestimated banana density in areas where bananas were sparsely grown or when intercropping was common. For instance, the largest discrepancy was observed in the Mtwara region, where the calculated area was 8% compared with the reported area of 0.1%. Mtwara is Tanzania’s leading cashew nut producer, contributing to approximately 70% of the national output (Lukurugu et al., 2022). Other perennial crops cultivated in these regions include mangoes, oil palm, and oranges. We normalized banana production using Equation 2 in each region using data on the total area planted with bananas (monoculture plus mixed) from the Tanzania National Sample Census of Agriculture 2019–2020 (National Sample Census of Agriculture, 2019/2020). Census data showed that a small fraction of land was planted with bananas in Tanzania. Three regions (Dar es Salaam, Kagera, and Kilimanjaro) had more than 1% of their area planted with bananas (**Figure 1D**).

For our derived maps at 1 km resolution, the mean percentage area planted with bananas is between 1.1 × 10^−4^% and 42% (**Figure 1E**), the minimum percentage area planted with bananas is between 0% and 26% (**Figure 1F**), and the maximum percentage area planted with bananas is between 4.8 × 10^−4^% and 49% (**Figure 1G**). We plotted a boxplot of the percentage area planted with bananas for the 100 grid cells with the largest banana production in **Figure 1H**. Only a few grid cells had bananas occupying more than 20% of the area; these were in the Kagera and Kilimanjaro regions.

#### 3.1.2 Ogun State, Nigeria

**Figure 2:**
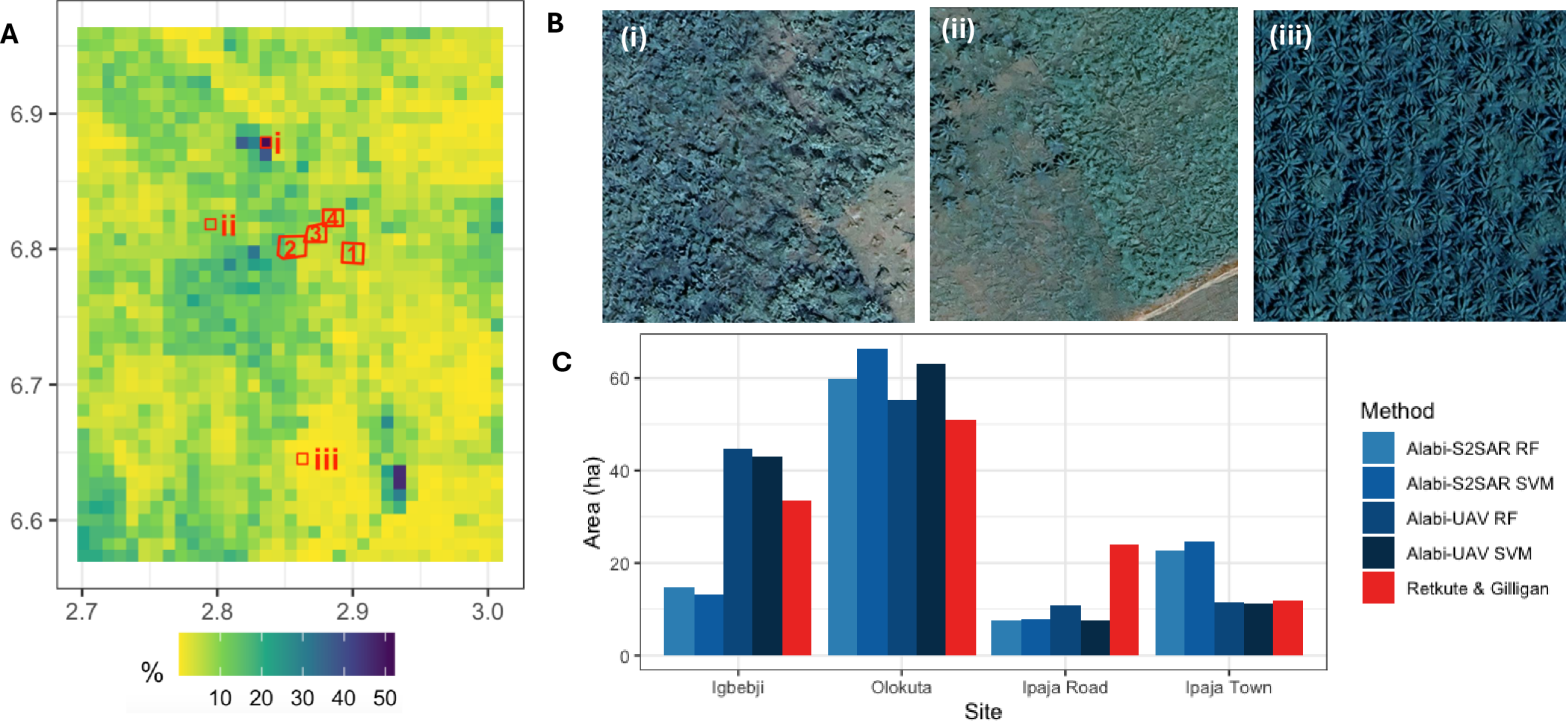
Constructing banana production map for Ogun State. **(A)** Fraction of a grid cell occupied by bananas. The four UAV flight sites from Alabi et al. (2022) (red outlines) were (1) Igbeji, (2) Olokuta, (3) Ipaja Road, and (4) Ipaja Town. **(B)** Three examples with different banana production levels: (i) high (ii) medium and no presence of bananas. The corresponding grid cells are shown in **(A)**, with indices i, ii, and iii. **(C)** Comparison with banana area estimated by Alabi et al. (2022). The corresponding polygons are shown in **(A)**, with indices 1, 2, 3, and 4. Satellite images in **(B)** were obtained from Google Earth Engine (Imagery @2024 Airbus, CNES/Airbus, Landsat/Copernicus, Maxar Technology).

The algorithm for constructing banana production maps was further tested using data from Ogun State. We combined data on vegetation, canopy height, and built-up areas with a mask derived from the oil palm spatial data (Descals et al., 2021). This approach addresses the lack of crop statistics on agricultural production at the administrative unit level. A map of banana production is shown in **Figure 2A**. The spatial distribution was highly heterogeneous, with the percentage of grid cells occupied by bananas ranging from 0% to 52.2%. Satellite images showing high banana production (∼50%), medium banana production (∼ 20%), and no banana production are shown in **Figure 2B**. The latter corresponds to the area planted with oil palms.

We calculated the area occupied by bananas at the same four sites as in Alabi et al. (2022): Igbebji, Olokuta, Ipaja Road, and Ipaja Town. We found good agreement between the estimated areas occupied by bananas based on our method and estimated banana areas based on UAV and Sentinel 2 + SAR data (**Figure 2C**). There was variability in estimates based on data acquisition and ML technique in Alabi et al. (2022). Our estimated area was between the UAV and Sentinel 2 + SAR predictions for the Igbebji and Ipaja Town sites, lower than the ML-based estimates for Olokuta, and higher than ML-based estimates for Ipaja Road. The correlation was 0.915 between our estimates and those estimates based on Random Forest + UAV data (UAV RF), 0.927 between our estimates and estimates based on Support Vector Machine + UAV data (UAV SVM), 0.726 between our estimates and estimates based on Random Forest + Sentinel 2 data + SAR data (S2SAR RF), and 0.704 between our estimates and those based on Support Vector Machine + Sentinel 2 data + SAR data (S2SAR SVM).

### 3.2 Model parameterization and simulations

**Figure 3:**
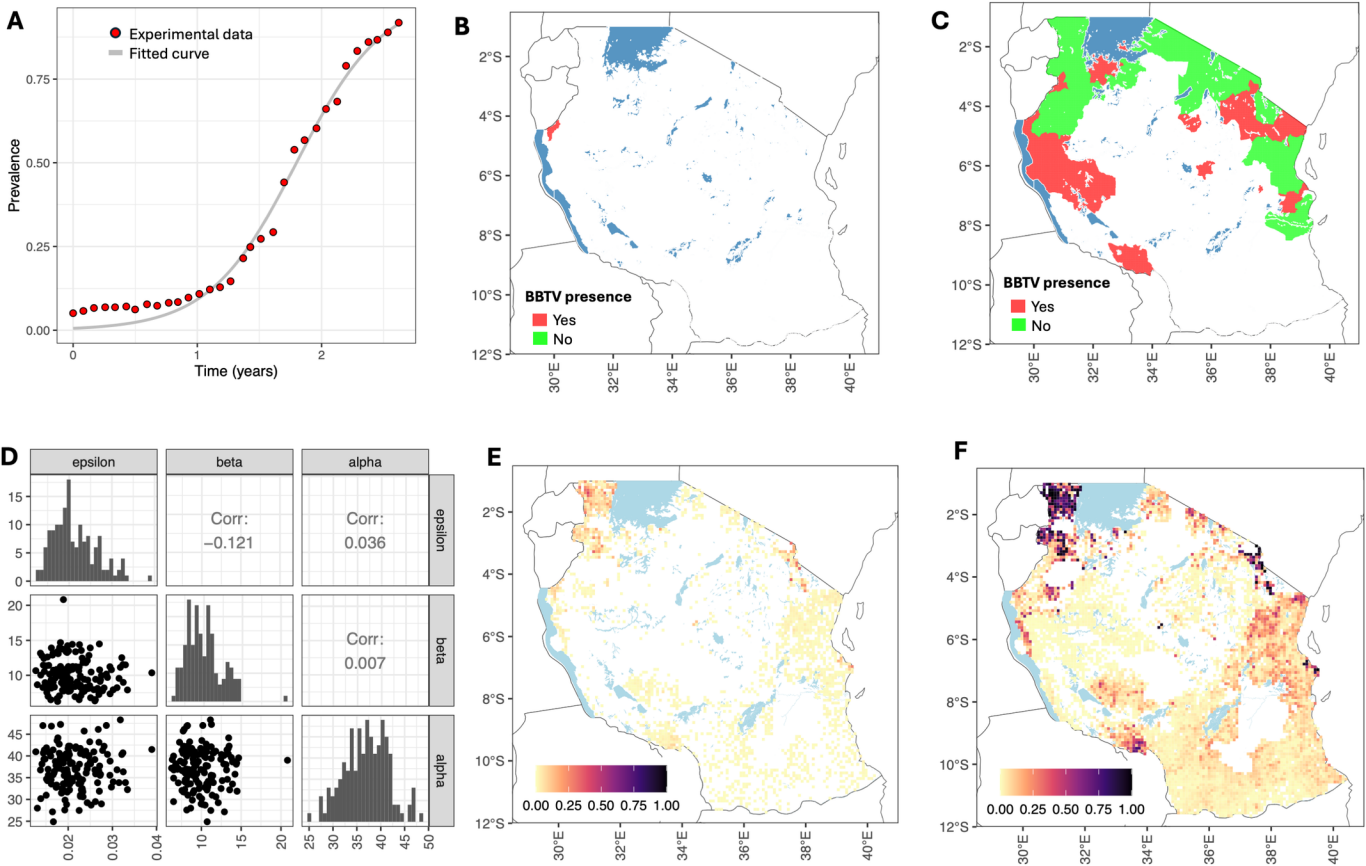
Model parameterization and simulations. **(A)** Progression of the fraction of infected hosts over time within the cell: data from Omondi et al. (2020) (red dots) and the fitted logistic equation (gray lines). **(B)** Survey results for 2020 (Shimwela et al., 2022). Districts with a BBTV presence are indicated in red. **(C)** Survey results conducted in 2023 (Mahuku and Kumar, 2023). Districts with a BBTV presence are shown in red, and districts with a BBTV absence are shown in green. **(D)** Posterior distribution of primary infection rate (*ϵ*), secondary infection rate (*β*), and dispersal scale (*α*). **(E)** Simulated pattern of BBTV transmission in 2023. **(F)** Simulated pattern of BBTV transmission by 2030.

The progression of the fraction of infected hosts within the grid cell over time was assumed to be described by a logistic equation given by Equations 3. The experimental data from Omondi et al. (2020) and the fitted logistic curve are shown in **Figure 3A**. The estimated values are *r* = 2.86 year^−1^ and *p*_0_ = 0.006.

The BBTV spread between the grid cells given by Equations 4, 5, was parameterized using data from two surveys conducted in 2020 and 2023. We aggregated the survey results at the district level, i.e., we classified a district as having BBTV presence if at least one survey found BBTV-positive samples. The spatial distributions of districts with and without BBTV presence are shown in **Figures 3B, C**). We sampled 10^6^ parameter sets from a uniform prior distribution *log*10(*ϵ*) ∼ *U*[−6,3], *log*10(*β*) ∼ *U*[−6,3] and *α* ∼ *U*[0.1,1,000]. We seeded BBTV infections in two locations: (i) at a randomly sampled grid cell in Buhigwe district (corresponding to cross-border introduction of BBTV), and (ii) at a randomly sampled grid cell in the Tanga region (correspondings to bringing in infected planting material). We retained the top 0.1% of parameters with the highest scores, as determined by Equation 6, yielding *M_threshold_* = 0.83. The posterior distributions of the parameters are shown in **Figure 3D**. The range for the primary infection rate was 0.013 day^−1^–0.039 day^−1^, the secondary infection rate was 5.7 day^−1^–20.8 day^−1^, and the dispersal scale was 25 km–48 km.

Using the estimated parameters, we simulated the pattern of BBTV transmission in 2023 and 2030 (**Figures 3E, F**). The score metric value for 2020 was 0.985, and that for 2023 was 0.851. A map for the predicted extent of infection in 2030 confirms that the key areas requiring focused attention for surveillance and preventive measures to control BBTV spread are areas with high banana production, i.e., Kagera, Kilimanjaro, Dar es Salaam, and Mbeya regions.

## 4 Discussion

This study presents the first comprehensive stochastic model that outlines methods for developing data-driven models to generate banana production maps, and process-based models to evaluate the potential spread of BBTV on a landscape scale. Few studies have been conducted to model the spread of BBTV at a plantation or field scale (Allen and Barnier, 1977; Allen, 1987; Smith et al., 1998; Varghese et al., 2020). Plantation-scale models provide tools for optimizing control of commercial farms. Recently, regression-based methods have been applied to identify areas that have high environmental suitability for BBTV establishment, such as Uganda (Ocimati et al., 2024) and the entire African continent (Bouwmeester et al., 2023). However, maps showing suitability for BBTV cannot be used to inform smallholder farmers of the best practices for mitigation facing the threat of the imminent spread of BBTV.

Our methodology for mapping banana cultivation showed good agreement with the results based on machine learning combined with high-resolution RGB and multispectral aerial imagery conducted in Ogun State in Nigeria (Alabi et al., 2022). Besides banana and plantain, a variety of crops are cultivated in the state, including cocoa, oil palm, oranges, maize, cassava, cowpea, and vegetables, with farmers predominantly practicing intercropping (Alabi et al., 2022). We found a high degree of correlation between the estimated banana area based on the approach introduced in this study and the estimates from ML and UAV images (*ρ* = 0.915–0.926). Pixel-based classifications and ML models require the manual delineation and annotation of thousands of reference points (Gomez Selvaraj et al., 2020). For example, at the Olokuta site, 223,578 georeferenced polygons were derived for different classes (Alabi et al., 2022). Therefore, because of the amount of effort required to obtain and process UAV-derived imagery, it is not possible to scale this method to the national level. The proposed workflow for map construction can be deployed at the country level, as demonstrated in the current study. We used publicly available data to derive a high-resolution banana production map. In contrast, there is no repository of UAV-derived imagery.

Our methods require the distribution of canopy height and NDVI values typical for bananas as input. We used photo-interpretation of high-resolution satellite imagery from Google Earth Pro to sample locations and derive these distributions. The range for canopy height was between 2 m and 6 m, and the annual median NDVI was in the range of ∈ [0.55,0.88]. Many factors can influence banana plant height, such as stage of growth (Tixier et al., 2004), cultivar (Daniells and O’Farrell, 1988), planting density, and irrigation (zum Felde et al., 2016). Plant photosynthetic activity also has a complex dependence on seasonality and environmental variables (Brown and de Beurs, 2008). The wide ranges of canopy height and annual median NDVI distributions that we obtained reflect this variability. However, the methodology allowed us to derive a probabilistic host distribution, which to the best of our knowledge, has not been performed before.

Our methodology has several limitations. First, it requires a large amount of data (**Table 1**). Another aspect is that the algorithm performed well in areas with high banana presence (i.e., in the Kagera region), but overestimated banana density in areas where banana was grown sparsely or where intercropping was common. The performance of our method depended on the type of intercropping used. When bananas are grown in association with annual food crops, such as maize, rice, and cassava, we expect reasonable efficiency (as seen in **Figure 2B**) The efficiency will be low when bananas are combined with perennial trees. For example, oil palm trees have a morphology similar to that of bananas. In Tanzania, the Kigoma region has the largest harvested area of oil palm (4,726 ha), followed by Mbeya (1,614 ha), whereas the least harvested area of oil palm is in Kagera (2 ha) (National Sample Census of Agriculture, 2019/2020). There is also a diverse variety of permanent tree crops cultivated across various regions of Tanzania (Kilawe, 2024), with fruit trees present on almost every farm (Delobel et al., 1991). To overcome this limitation, we normalized the total area planted with bananas in each region using data from the Tanzania National Sample Census of Agriculture 2019–2020 (National Sample Census of Agriculture, 2019/2020). However, the use of national data to address overestimation in areas with low production may face challenges in regions where the data quality is poor or entirely lacking within a country or production landscape. Additional information, such as the locations of oil palms, can be utilized to mask such areas, as in the case of Ogun State.

We introduced a novel parameter estimation method by proposing a score metric that assesses the similarity of simulated outbreaks to survey data at the district level. To parameterize the model for BBTV spread in Tanzania, we used the survey results from 2020 (Shimwela et al., 2022) and 2023 (Mahuku and Kumar, 2023) aggregated at the district level. To account for the variability in district areas, we normalized the contribution of each district by the total number of grid cells within a district. This score also accounts for stochasticity in the model outputs. The range of permissible values of the score metric lie between zero and one, where one corresponds to simulations reproducing survey results exactly at the district level. For the model simulations using the fitted parameters, we obtained *M*_2020_ = 0.985 and *M*_2023_ = 0.851. Another novel aspect of the ABC rejection algorithm we used is maximizing the score metric instead of minimizing a summary statistic.

A key assumption in demonstrating the approach to modeling BBTD spread in Tanzania was that the disease was confined to the districts in which it had been reported during the surveys. Another key aspect of this approach is model validation. Only a few studies have validated the spatial spread of crop pathogens and pests on a national scale, which requires high-resolution spatial and temporal surveillance data (Nguyen et al., 2023; Godding et al., 2023; Retkute et al., 2024). These two aspects highlight the critical role of surveys, as data on the presence and absence of the disease at various epidemic stages are essential not only for disease management, but also for accurate prediction. Regular proactive surveillance plays a key role in providing updated data on the current status of the epidemic spread for use in initiating model predictions of future spread. Second, updated surveillance provides an invaluable resource from which to update model parameterizations and validate models by comparing model predictions with ground-based observations.

Our results have important implications for BBTD management. In Tanzania, bananas are a vital food and commercial crop and serve as a major source of raw materials for the beverage and handicraft industries (Luzi-Kihupi et al., 2015), with almost two million households involved in banana production activities (National Sample Census of Agriculture, 2019/2020). Banana is an important primary staple crop, with annual banana consumption reaching 500 kg–1,500 kg per head in southeastern Kilimanjaro (Yamaguchi and Araki, 2004). Simulated patterns of BBTV transmission in Tanzania emphasize the key areas requiring focused attention for combating BBTV through surveillance and preventive measures to control its spread, i.e., Kagera, Kilimanjaro, Dar es Salaam, and Mbeya. Future work will focus on using the BBTV transmission model to evaluate control options, including roguing, impact of clean seed production networks, and clean seed deployment.

## Data Availability

The data and code to produce a banana production map are available on a GitHub repository at https://github.com/rretkute/BananaProductionMapping.

## Supplementary material

Supplementary Figure 1 Percentage of area planted with banana in Tanzania regions based on data from the Census of Agriculture and calculated (not adjusted) using the proposed methods. Data are grouped according to the presence of perennial crops: banana, cashew nut, mango, oil palm, and orange. Source: (National Sample Census of Agriculture, 2019/2020).
