# Supplementary figures and images for: Developing a spatio-temporal model for banana bunchy top disease: leveraging remote sensing and survey data
Source: Front Plant Sci. 2025 Jun 9;16:1521620. doi: 10.3389/fpls.2025.1521620 (PMC12183257; doi:10.3389/fpls.2025.1521620)

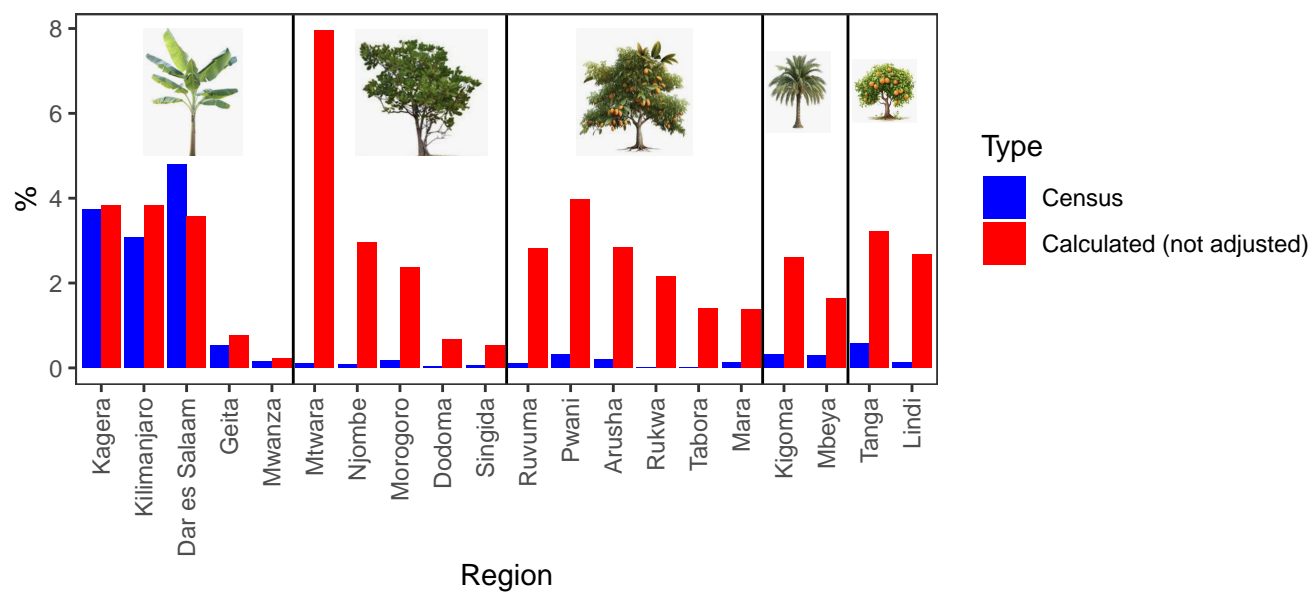

Supplement: Supplementary Figure 1 — Percentage of area planted with banana in Tanzania regions based on data from the Census of Agriculture and calculated (not adjusted) using the proposed methods. Data are grouped according to the presence of perennial crops: banana, cashew nut, mango, oil palm, and orange. Source: (National Sample Census of Agriculture, 2019/2020). [file Image1.pdf]
